# The Association between Social Support and Attitude toward Technology Differs by Educational Level

**DOI:** 10.1093/geroni/igab046.3347

**Published:** 2021-12-17

**Authors:** Susanna Joo, Changmin Lee, Kwang Joon Kim, DaeEun Kim, Hey Jung Jun

**Affiliations:** 1 Yonsei University, Seodaemun-gu, Seoul-t'ukpyolsi, Republic of Korea; 2 Yonsei University, Seoul, Seoul-t'ukpyolsi, Republic of Korea; 3 Severance Hospital, Yonsei University College of Medicine, Seoul, Seoul-t'ukpyolsi, Republic of Korea

## Abstract

The present study aimed to examine the moderating effects of the educational context on the association between social support from family members and attitude toward using gerontechnology among Korean older adults. The sample was Korean older adults without dementia (N=310, Age: 65-89, M=70.18, SD=4.58). Data were collected by online recruiting in February 2021. The dependent variable was the attitude toward using gerontechnology, especially, an exoskeleton robot for exercise. Independent variables were four types of social support (emotional, instrumental, physical, and financial support) from family members. Moderating variable was the binary educational group (high school and below, or college and over). We analyzed four regression models including each interaction term between education and a type of social support using PROCESS macro and bootstrapping. Results showed educational context had a single significant moderating effect on the association between emotional support and attitude toward using gerontechnology. Specifically, emotional support had a significant effect on having a positive attitude toward using gerontechnology among older adults who graduated high school or were less educated. However, it was not significant among older adults who were highly educated. Moreover, other types of social support did not have significant main effects as well as interaction effects with education on the attitude toward using gerontechnology. Findings of the present study implied emotional support from family members, such as spouse, children, or siblings, was useful to enhance having a positive attitude toward using new technology, especially for older adults who did not experience college-level educational context.

